# High-Level Serum Fibroblast Growth Factor 21 Concentration Is Closely Associated With an Increased Risk of Cardiovascular Diseases: A Systematic Review and Meta-Analysis

**DOI:** 10.3389/fcvm.2021.705273

**Published:** 2021-08-26

**Authors:** Yucong Zhang, Jinhua Yan, Ni Yang, Zonghao Qian, Hao Nie, Zhen Yang, Dan Yan, Xiuxian Wei, Lei Ruan, Yi Huang, Cuntai Zhang, Le Zhang

**Affiliations:** Department of Geriatrics, Institute of Gerontology, Tongji Hospital, Tongji Medical College, Huazhong University of Science and Technology, Wuhan, China

**Keywords:** fibroblast growth factor 21, coronary heart disease, hypertension, cardiovascular disease, risk factor, meta-analysis

## Abstract

**Background:** The association between fibroblast growth factor 21 (FGF21) and cardiovascular disease (CVD) risk remains unclear. We conducted this systematic review and meta-analysis to evaluate the association between FGF21 and CVDs, and relevant vascular parameters.

**Methods:** PubMed and Web of Science databases were systematically searched to identify relevant studies published before March 2021. The FGF21 concentration was compared between individuals with and without CVDs. The effect of FGF21 on CVD risk was assessed by using hazard ratio (HR) and odds ratio (OR). The association between FGF21 and vascular parameters was assessed by Pearson's *r*. Study quality was assessed using Newcastle–Ottawa Scale and Joanna Briggs Institution Checklist.

**Results:** A total of 29,156 individuals from 30 studies were included. Overall, the serum FGF21 concentration was significantly higher in CVD patients (*p* < 0.001), especially for coronary artery disease (CAD) (*p* < 0.001) and hypertension (*p* < 0.001). The pooled OR (*p* = 0.009) and HR (*p* < 0.001) showed that the risk of CVDs increased with FGF21. The linear association between FGF21 and vascular parameters, including pulse wave velocity (*r* = 0.32), carotid intima-media thickness (*r* = 0.21), ankle-brachial index (*r* = 0.33), systolic blood pressure (*r* = 0.13), and diastolic blood pressure (*r* = 0.05), was insignificant. The incidence of overall CVDs (*p* = 0.03) was significantly higher in individuals with higher FGF21 levels.

**Conclusion:** High-level serum FGF21 concentration is closely associated with an increased risk of CVDs, which may be independent of vascular parameters. A standard FGF21 classification threshold needs to be established before clinical use for CVD risk assessment.

**Systematic Review Registration:**https://www.crd.york.ac.uk/prospero/display_record.php?RecordID=241968, identifier: CRD42021241968.

## Introduction

Cardiovascular diseases (CVDs) are the leading cause of death globally and are composed of heart and blood vessel diseases. According to data from the World Health Organization, CVDs caused 17.9 million deaths in 2016, representing 31% of all global deaths (1). Early identification of risk factors to prevent or treat CVDs is very important for reducing morbidity. Various biomarkers have been investigated for their roles in the diagnosis and prognosis of CVDs, including blood lipids, blood glucose, weight, and age (2).

Fibroblast growth factors (FGFs) are a family of signaling proteins, in which FGF21 is a metabolic regulating hormone in energy homeostasis. Due to the lack of a heparin binding domain, FGF21 can be released in the circulation and function in an endocrine manner (3, 4). Because of its ability to regulate carbohydrate and lipid metabolism, FGF21 is considered to have multiple beneficial effects on major cardiovascular risk factors, such as hyperlipidemia, obesity, and diabetes (2). In addition, an increasing number of studies evaluated the potential role of FGF21 as a CVD biomarker. Among them, some studies reported a significant association between FGF21 and CVDs, while others found the association insignificant (5). Therefore, we conducted this systematic review and meta-analysis to evaluate the association between FGF21 and CVDs, especially for those closely related to metabolic abnormalities including coronary heart disease (CHD) or coronary artery disease (CAD), atrial fibrillation, cerebral infarction, and hypertension (2). We also evaluated the relationship of FGF21 with vascular parameters, including carotid intima-media thickness (cIMT), pulse wave velocity (PWV), ankle-brachial index (ABI), and blood pressure (BP).

## Materials and Methods

This work was executed in accordance with the Preferred Reporting Items for Systemic Reviews and Meta-analysis (PRISMA) guidelines (6). It was also registered in the International Prospective Register of Systematic Reviews (PROSPERO) before screening studies for inclusion (ID: CRD42021241968).

### Literature Search

We conducted a systematic literature search by searching PubMed and Web of Science in March 2021. Studies that assessed the association between serum FGF21 concentration and CVDs and relative vascular parameters were identified through full-text review. The following terms and their combinations were employed: “fibroblast growth factor 21,” ”FGF21,” “coronary artery disease,” “coronary heart disease,” “artery stiffness,” “aortic aneurysm,” “hypertension,” “blood pressure,” ”pulse wave velocity,” “atherosclerosis,” “ischemic heart disease,” “cerebral infarction,” “myocardial infarction,” “angina,” “atrial fibrillation,” “cardiovascular,” ”cardiac,” ”cardio-ankle vascular index,” “carotid intima-media thickness,” “ankle-brachial index,” and “flow-mediated dilatation.”

### Selection Criteria

The inclusion criteria were as follows: (1) studies that assessed the association between serum FGF21 concentration and CVDs, including CHD or CAD, atrial fibrillation, cerebral infarction and hypertension, or relative vascular parameters, including cIMT, PWV, ABI, BP, cardio-ankle vascular index, and flow-mediated dilatation; (2) the results contained at least one set of the following statistics: (a) mean with standard deviation or median with quartile of serum FGF21 concentration in patients with or without CVDs, (b) odds ratios (ORs) or hazard ratios (HRs) with corresponding 95% confidence intervals (CIs) of serum FGF21 concentration and incidence of CVDs, (c) Pearson correlation coefficient (Pearson's *r*) of serum FGF21 concentration and vascular parameters, and (d) incidence of CVDs in individuals with different serum FGF21 levels; and (3) adult clinical studies that were published in English.

The exclusion criteria were as follows: (1) studies that were reviews, letters, meeting abstracts, case reports, commentary, or editorials; (2) studies that only reported on rheumatic heart disease, cardiomyopathy, or microvascular disease, and not CHD, atrial fibrillation, cerebral infarction, or hypertension; (3) duplicate studies with overlapping data; (4) studies that reported invalid data that could not be pooled; and (5) studies on pregnant women.

According to the selection criteria, the initial screening of studies was based on titles and abstracts. Then, the full texts of the potential studies were assessed. An additional manual search of references from identified studies was also performed. All studies were independently screened by two reviewers (YZ and JY). A third researcher (NY) was consulted to resolve disagreements.

### Data Extraction and Quality Assessment

Two reviewers independently extracted data from the included studies. Basic information and patient baseline characteristics of all studies were extracted. To assess the association between serum FGF21, CVDs, and vascular parameters, the following data were extracted: (1) mean with standard deviation or median with quartile of serum FGF21 concentration; (2) ORs or HRs with corresponding 95% CIs; (3) Pearson's *r*; and (4) incidence of CVDs.

Quality assessment was independently performed by two reviewers (ZQ and HN). Discrepancies were resolved by discussion with a third reviewer (ZY). The quality of cohort studies and case–control studies was assessed by using the Newcastle–Ottawa Quality Assessment Scale (NOS) (7). Studies scoring > 5 were considered to be high-quality. The quality of cross-sectional studies was assessed by using the Joanna Briggs Institution (JBI) Checklist for Analytical Cross-Sectional Study (joannabriggs.org/research/critical?appraisal-tools.html). Publication bias was assessed by funnel plots if the number of included cohorts was ≥10. Publication bias was considered to be significant if the funnel plot was asymmetric.

### Data Analysis

The mate analysis was performed by using RevMan 5.3 (the Nordic Cochrane Center, Copenhagen, Denmark). To achieve conservative results, a random-effects model was employed for pooled analysis. Heterogeneity was tested by using the Chi-squared test and *I*^2^ statistic. *p* < 0.05 or *I*^2^ > 50% indicated that the heterogeneity was significant. The overall effects were determined by the *Z*-test, and *p* < 0.05 was considered statistically significant. Subgroup analysis was conducted according to specific CVDs or disease outcomes.

Due to the differences in the number of FGF21 levels among studies, we chose to extract the CVD incidence from the individuals with the highest or lowest FGF21 level in each study.

Medians with quartiles were transformed into means with standard deviations for pooled estimates by using the webpage tool in the BOX-COX manner developed by McGrath et al. (8).

Pearson's *r* was transformed into Fisher's *Z*-value for pooled estimates. The resulting value was then weighted with the inverse of the variance of the correlation coefficients. The 95% CI of the pooled weighted Fisher's *Z* coefficients was also calculated, after which all of the values were back-transformed into *r* using the following formula (9). The linear association was considered to be very high, high, moderate, low, and irrelevant when summary |*r*| was larger than 0.8, between 0.6 and 0.8, between 0.4 and 0.6, between 0.2 and 0.4, and smaller than 0.2, respectively.

$$\begin{array}{ll} \text{Fishe}{\text{r}}^{\prime }\text{s Z} & =0.5\;\times \ln\frac{1+\text{r}}{1-\text{r}}\\ v_{z} & =\;\frac{1}{n-3}\\ S_{E} & =\;\sqrt{v_{z}}\\ \text{Summary }r & =\;\frac{e^{2z}-1}{e^{2z}+1\;} \end{array}$$

## Results

**Figure 1:**
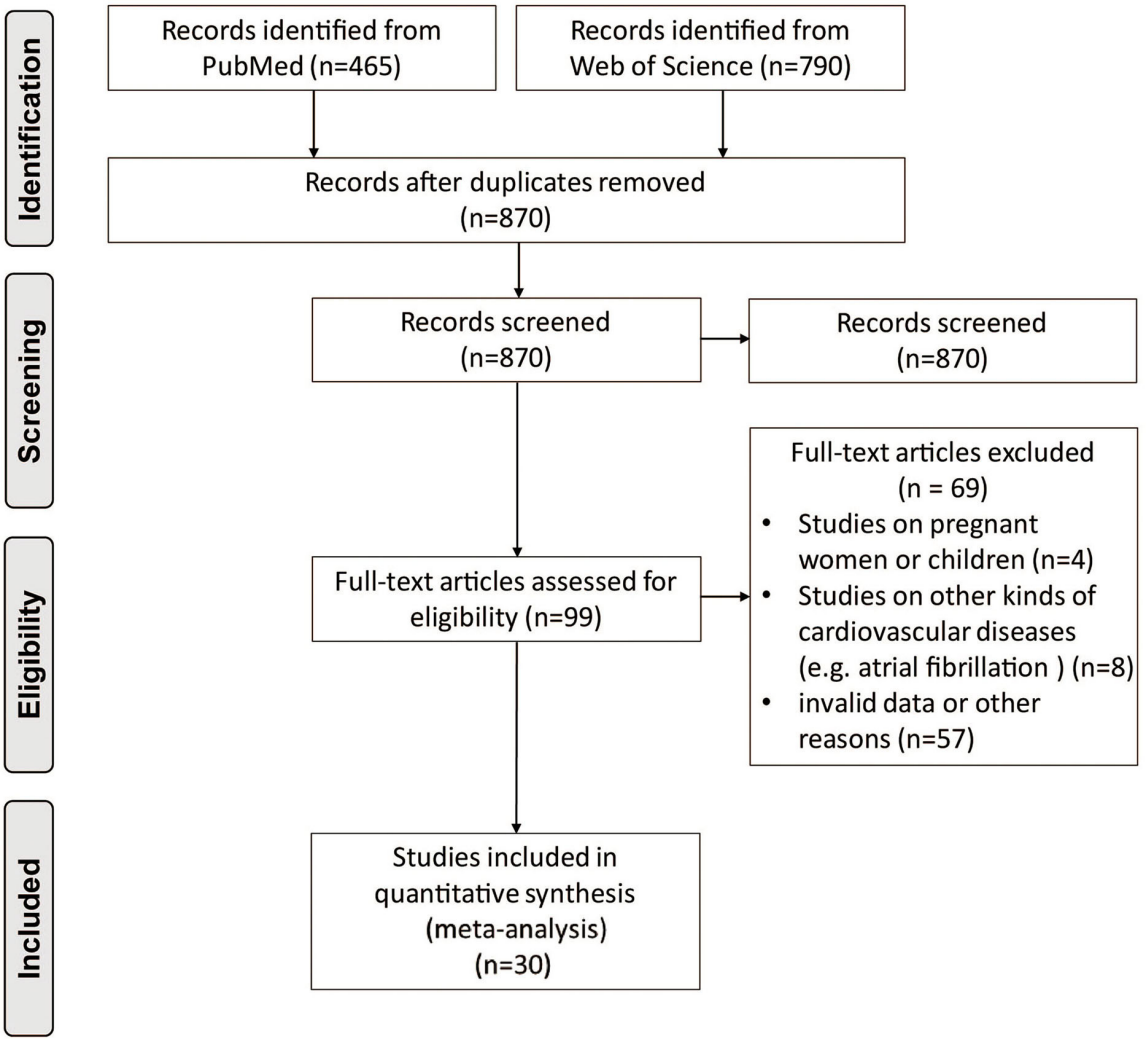
Preferred reporting items for systemic reviews and meta-analysis flow diagram of literature screening.

After removing duplicate articles, 870 articles were identified in the initial database search. After screening titles and abstracts, 99 articles remained for further full-text evaluation. Finally, 30 articles with 29,156 individuals were included in the meta-analysis (10–39). The flow diagram of study screening is shown in Figure 1. Tables 1–4 summarize the basic information and patient baseline characteristics of these studies. Supplementary Table 1 summarizes the patient selection criteria of these studies. Among these studies, 14 reported serum FGF21 concentration in patients with or without CVDs (10–23), 14 reported ORs or HRs of serum FGF21 concentration and incidence of CVDs (11, 14, 17, 18, 20, 24–32), 8 reported Pearson's *r* of serum FGF21 concentration and vascular parameters (12, 15, 16, 25, 30, 33–35), and 6 reported incidence of CVDs in individuals with different serum FGF21 levels (27, 30, 36–39).

**Table 1:** Baseline characteristics of studies reported serum FGF21 concentration in patients with or without CVDs.

**References**	**Region**	**Study design**	**Disease**	**CVDs group (*n*)**	**Control group (*n*)**	**Male (%)**		**Age (years)** **^#^**		FGF21 concentration (pg/ml)^*^	
						**CVDs**	**Control**	**CVDs**	**Control**	**CVDs**	**Control**
Wu et al. (10)	China	Cross-section	Stable CAD	116	45	72.4	71.1	63.4 ± 9.0	61.2 ± 8.7		
Wu et al. (11)^##^	China	Cross-section	Developed IHD	36	608	55.6	38.8	71.6 ± 14.1	57.6 ± 13.5	479.5 (302.4–627.0)	325.2 (189.0–498.9)
			Atherosclerosis	186	185	61.8	51.4	58.6 ± 8.4	54.4 ± 9.0	266.7 (135.5–415.2)	198.4 (99.9–373.6)
Basurto et al. (13)^##^	Mexico	Cross-section	Subclinical atherosclerosis	75	65	0	0	53.0 (49.0–61.0)^*^	50.0 (46.0–53.0)^*^	127.0 (46.9–200.0)	185.2 (95.0–260.4)
Chen et al. (14)^##^	China	Prospective cohort	AMI	183	55	84.7	60.0	63.6 ± 10.9	66.9 ± 9.5	143.8 (75.2–254.3)	121.0 (57.1–179.6)
Cheng et al. (15)^**^	China	Cross-section	stable angina pectoris	66	55	54.6	38.2	60.7 ± 9.5	59.4 ±9.2		
Trakarnvanich et al. (16)^**^	Thailand	Cross-section	Cardiovascular events	12	78	64.4		47.2 ±11.8			
Lee et al. (17)	China	Prospective cohort	Incident CHD	147	3,381	66.7	51.1	65.6 ± 12.1	60.6 ± 12.8	222.7 (92.8–438.4)	151.1 (75.6–274.6)
Zhang et al. (18)^##^	China	Cross-section	AMI	55	45	81.0	56.3	64 ± 11	63 ± 10	25 (16–34)	14 (11–20)
Kim et al. (19)^##^	Korea	Cross-section	CAD without diabetes	30	30	70.0	76.7	60.5 ± 11.0	58.5 ± 10.4	277.1 (155.6–476.6)	141.7 (73.9–180.9)
			CAD with diabetes	30	30	20.0	30.0	61.7 ± 11.0	63.1 ± 10.4	278.5 (190.5–875.1)	224.5 (146.8–337.4)
Semba et al. (22)^##^	USA	Case-control	Hypertension	235	509	54.5	47.7	–	–	269 (161–457)	208 (117–335)
Shen et al. (20) ^##^	China	Cross-section	CAD without NAFLD	136	47	72.1	48.9	68.2 ± 9.9	64.6 ± 9.9		
			CAD with NAFLD	43	27	62.8	58.3	65.5 ± 10.9	61.3 ± 7.7		
Chow et al. (12)^##^	China	Cross-section	Hypertension	363	307	45.2	39.4	58.2 ± 12.9		283.0 (175.8–455.3)	197.3 (126.2–330.3)
Lee et al. (21)	Korea	Retrospective cohort	CAD	60	129	57.1		50.3 ± 7.6	62.1 ± 9.8		
Lin et al. (23)	USA	Cross-section	CHD	135	61	49.6	50.8	69 ± 5.8	68.6 ± 10.8		

*FGF21, fibroblast growth factor 21; CVD, cardiovascular disease; CAD, coronary artery disease; IHD, ischemic heart disease; AMI, acute myocardial infarction; CHD, coronary heart disease; NAFLD, non-alcoholic fatty liver disease*.

# *Mean ± standard deviation*.

* *Median (interquartile range)*.

## *Original data was median with quartile*.

** *Data was transformed by the logarithm of 10*.

**Table 2:** Baseline characteristics of studies reported serum FGF21 concentration on the risk of CVDs.

**References**	**Region**	**Study design**	**Disease**	**Outcome**	**Cases (*n*)**	**Male (%)**	**Age (years)^**#**^**	**Median follow-up (range)**
Wu et al. (11)	China	Prospective cohort	Total ASCVD	HR	705	38.8	57.6 ± 13.5	74 months
			Subclinical atherosclerosis	OR	371	56.6	56.5 ± 8.9	–
Ong et al. (24)	Australia	Prospective cohort	Total CVD, hard CVD	HR	5,767	47.9	62.6 ± 10.2	14 years
Ong et al. (26)	Australia	Prospective cohort	MCVE	HR	1,992	80.1	61.3 ± 8.9	4.9 years
Yafei et al. (25)	Egypt	Cross-section	Subclinical atherosclerosis	OR	120	36.7	51.1 ± 7.7	–
Chen et al. (14)	China	Prospective cohort	MACE	HR	238	79.0	64.4 ± 10.7	24 months
Wu et al. (27)	China	Prospective cohort	MACE	HR	88	66.9	68.6 ±12.9	2.3 ± 1.3 years (mean ± SD)
Shen et al. (28)	China	Prospective cohort	MACE	HR	169	65.5	–	57 months
Shen et al. (29)	China	Prospective cohort	Cardiac death	HR	218	65.6	66.3 ± 10.1	5.0 years
Lee et al. (17)	China	Prospective cohort	Incident CHD	HR	3,528	51.8	60.8 ± 12.8	3.8 (2.8–5.0) years
Li et al. (30)	China	Prospective cohort	Cardiac death	HR	1,668	65.5		4.9 years
Zhang et al. (18)	China	Cross-section	AMI	OR	100	78.0	–	–
Xiao et al. (31)	China	Cross-section	Male subclinical atherosclerosis	OR	107	100	52.6 ± 9.7	–
			Female subclinical atherosclerosis	OR	105	0	55.6 ± 6.9	
Shen et al. (20)	China	Cross-section	CAD	OR	253	64.8	66.3 ± 10.1	–
Lenart-Lipinska et al. (32)	Poland	Prospective cohort	CVD	HR	87	51.7	61 (57–66)^*^	24 months
			CVD mobidity	HR				

*FGF21, fibroblast growth factor 21; ASCVD, atherosclerotic cardiovascular disease; CVD, cardiovascular disease; MCVE, major cardiovascular event; MACE, major adverse cardiovascular event; CHD, coronary heart disease; AMI, acute myocardial infraction; CAD, coronary artery disease*.

# *Mean ± standard deviation*.

* *Median (interquartile range)*.

**Table 3:** Baseline characteristics of studies reported linear association of serum FGF21 concentration and vascular parameters.

**References**	**Region**	**Study design**	**Vascular parameters**	**Male (%)**	**Age (years)^**#**^**	**Cases (*n*)**
Lee et al. (33)	Singapore	Cross-section	PWV	53.8	42.2 ± 15.8	78
Yamamoto et al. (35)	Japan	Cross-section	SBP, DBP	21	76.2 ± 8.0	73
Sunaga et al. (34)	Japan	Cross-section	SBP, DBP	79.6	70.5 ± 9.7	93
Yafei et al. (25)	Egypt	Cross-section	PWV, cIMT, ABI, SBP, DBP	36.7	51.1 ± 7.7	120
Cheng et al. (15)	China	Cross-section	SBP, DBP	55.3	60.1 ± 8.9	197
Trakarnvanich et al. (16)	Thailand	Cross-section	cIMT	64.4	47.2 ± 11.8	90
Li et al. (30)	China	Prospective cohort	SBP, DBP	65.5	63.5 ± 1.2	1,668
Chow et al. (12)	China	Cross-section	cIMT	42.5	58.2 ± 12.9	670

*FGF21, fibroblast growth factor 21; PWV, pulse wave velocity; SBP, systolic blood pressure; DBP, diastolic blood pressure; cIMT, carotid intima-media thickness; ABI, ankle-brachial index*.

# *Mean ± standard deviation*.

**Table 4:** Baseline characteristics of studies reported incidence of CVDs in individuals with different FGF21 levels.

**References**	**Region**	**Study design**	**Disease**	**Male (%)**	**Age (year)^**#**^**	**FGF21 level (pg/mL) classification and case (*n*)**
Gan et al. (36)	China	Prospective cohort	Hypertension, CHD (ACS and stent implantation)	62.9	54.2 ± 13.7	≤ 103.8 (283), 108.6–184.9 (283), 199.3–271.2 (283), ≥276.1 (283)
Wu et al. (27)	Australia	Prospective cohort	MI	66.9	68.6 ± 12.9	<113.7 (177), 113.7–227.3 (177), ≥227.3 (177)
Kohara et al. (37)	Japan	Cross-section	Total CVD	58.9	66.1 ± 12.9	1,029 (704–1,518) (44) 2,989 (2,184–5,973) (46)
Rusu et al. (38)	Romania	Prospective cohort	Total CVD	57.1	59.9 ± 12.5	<19.75 (17), ≥34.55 (18)
Ong et al. (39)	Australia	Prospective cohort	Total CVD	62.7	62.3 ± 6.9	<239.3 (3,232), 239.3–412.8 (3,233), ≥412.8 (3,232)

*FGF21, fibroblast growth factor 21; CHD, coronary heart disease; ACS, acute coronary syndrome; MI, myocardial infraction; CVD, cardiovascular disease*.

# *Mean ± standard deviation*.

According to NOS, all cohort studies and case–control studies were considered to be high quality (Supplementary Tables 2, 3). According to the JBI checklist, all cross-sectional studies were also considered to be high quality (Supplementary Table 4).

### Differences in Serum FGF21 Concentration in Individuals With or Without CVDs

**Figure 2:**
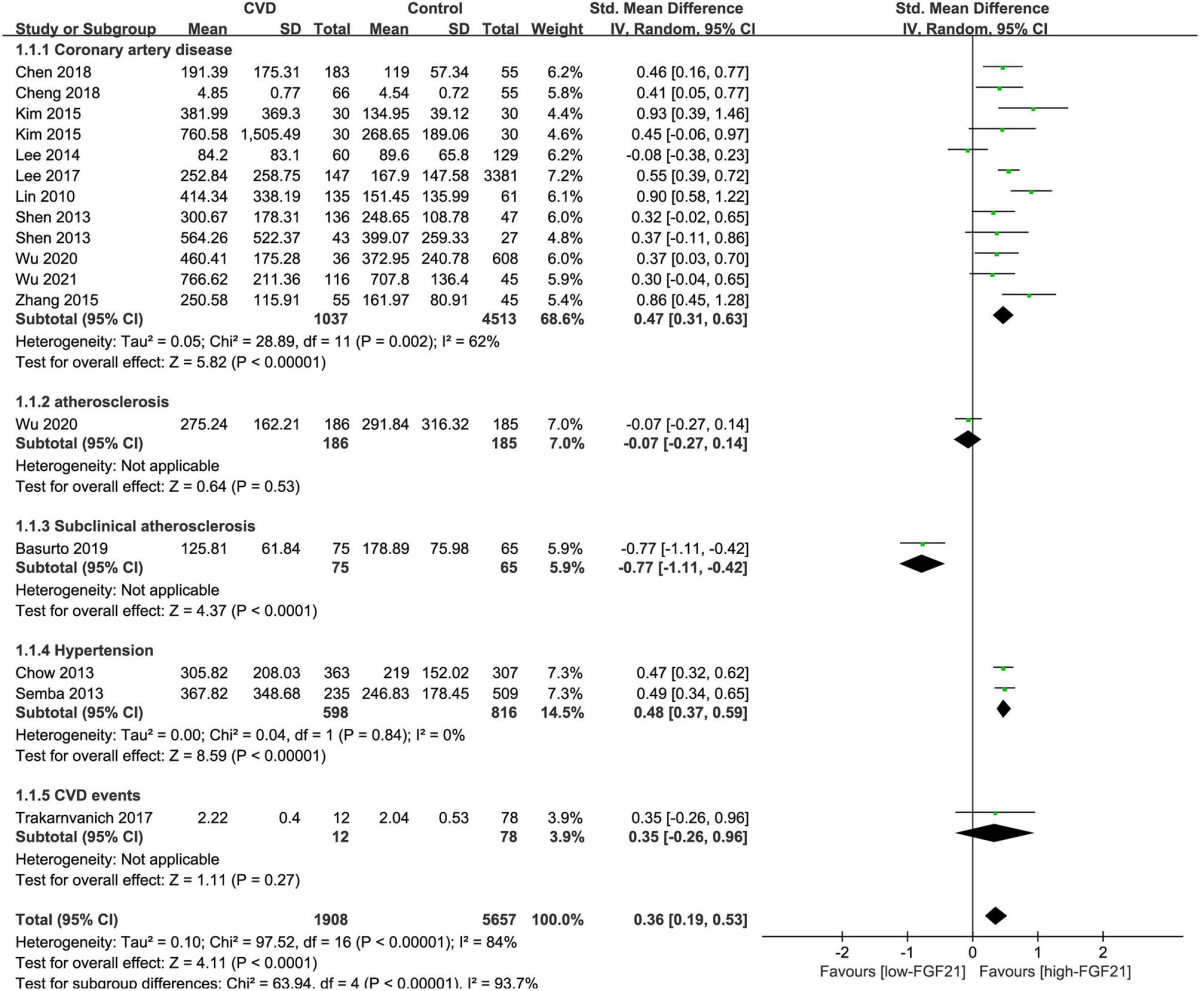
Forest plot of serum FGF21 concentration in individuals with or without CVDs. FGF21, fibroblast growth factor 21; CVD, cardiovascular disease. Mean value refers to the mean serum FGF21 concentration in each group.

The median serum FGF21 concentration in patients with or without CVDs is shown in Table 1. Three studies also provided serum FGF21 concentration of each patient in scatter plots (10, 14, 23) (Supplementary Figure 1). Among 14 studies, 9 studies reported that serum FGF21 concentration was significantly higher in patients with CVDs, including CAD and hypertension (11, 12, 14, 15, 17–19, 22, 23). However, one study reported that serum FGF21 concentration was significantly lower in patients with subclinical atherosclerosis (13). Overall, the serum FGF21 concentration was significantly higher in CVD patients than in those without CVDs [standard mean difference (SMD) = 0.58, 95% CI: 0.33–0.84, *p* < 0.001], especially for CAD (SMD = 0.75, 95% CI: 0.42–1.09, *p* < 0.001) and hypertension (SMD = 0.48, 95% CI: 0.37–0.59, *p* < 0.001) (Figure 2). The heterogeneity was significant among studies. Publication bias was assessed by a funnel plot, which indicated moderate publication bias (Supplementary Figure 2).

### Association Between Serum FGF21 Concentration and Prevalence of CVDs

**Figure 3:**
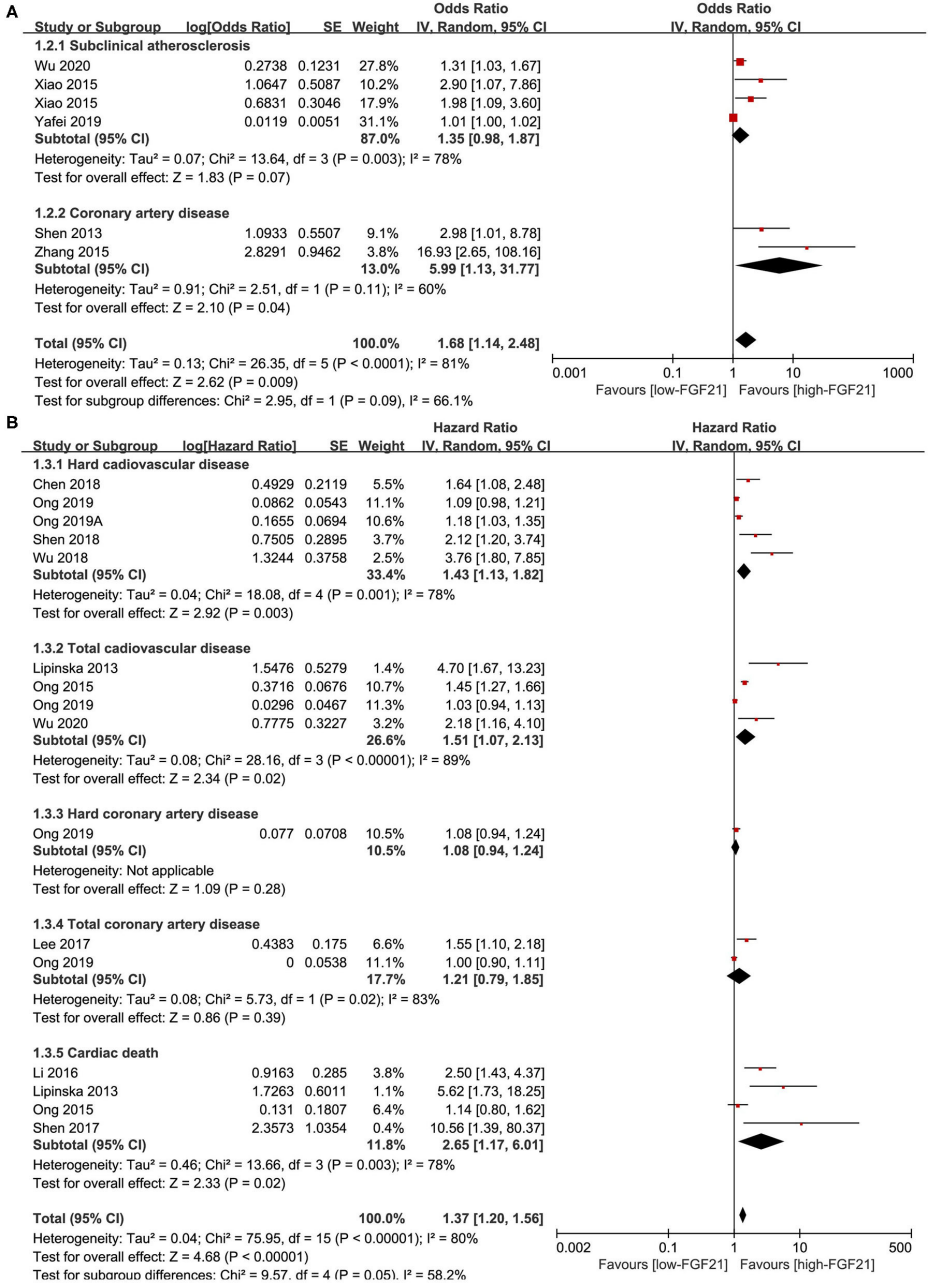
Forest plot of serum FGF21 concentration on the risk of CVDs. **(A)** Odds ratio; **(B)** Hazard ratio. FGF21, fibroblast growth factor 21; CVD, cardiovascular disease.

Five studies reported the ORs of FGF21 concentration and CVDs by logistic regression (Figure 3A). The pooled results showed that the risk of CAD (OR = 5.99, 95% CI: 1.13–31.77, *p* = 0.04) and overall CVDs (OR = 1.68, 95% CI: 1.14–2.48, *p* = 0.009) increased with FGF21. The heterogeneity was significant among studies.

Eleven studies reported the HRs of FGF21 concentration and CVDs (Figure 3B). The pooled results showed that the risk of hard CVD (HR = 1.43, 95% CI: 1.13–1.82, *p* = 0.003), total CVD (HR = 1.37, 95% CI: 1.20–1.56, *p* < 0.001), and cardiac death (HR = 2.65, 95% CI: 1.17–6.01, *p* = 0.02) increased with FGF21. The heterogeneity was significant among studies. Publication bias was assessed by a funnel plot, which indicated high publication bias (Supplementary Figure 3).

### Linear Association Between FGF21 and Vascular Parameters

**Figure 4:**
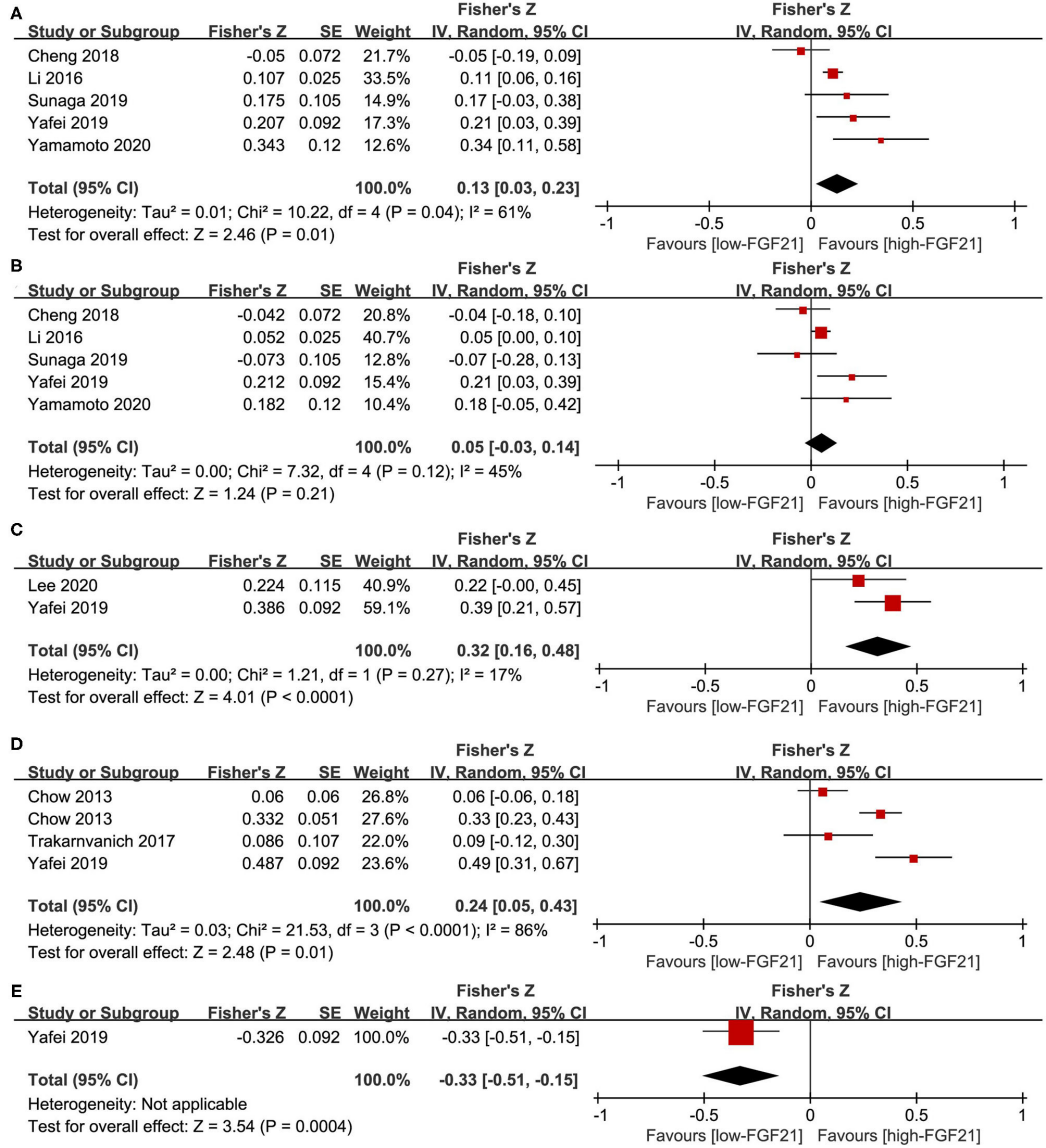
Forest plot of linear association between FGF21 and vascular parameters. **(A)** Systolic blood pressure; **(B)** diastolic blood pressure; **(C)** pulse wave velocity; **(D)** carotid intima-media thickness; **(E)** ankle-brachial index. FGF21, fibroblast growth factor 21. Linear association between FGF21 and vascular parameters was assessed by Pearson's *r*. Pearson's *r* was transformed into Fisher's *Z*-value for pooled estimates.

No association was observed between FGF21 and systolic BP (summary *r* = 0.13, Figure 4A) or diastolic BP (summary *r* = 0.05 Figure 4B). For the linear association between FGF21 and PWV, cIMT, and ABI, Fisher's *Z* was 0.32, 0.21, and −0.33, respectively (Figures 4C–E). The corresponding summary *r* values were 0.31, 0.24, and −0.31, which indicates a low association.

### Incidence of CVDs in Individuals With Different FGF21 Levels

**Figure 5:**
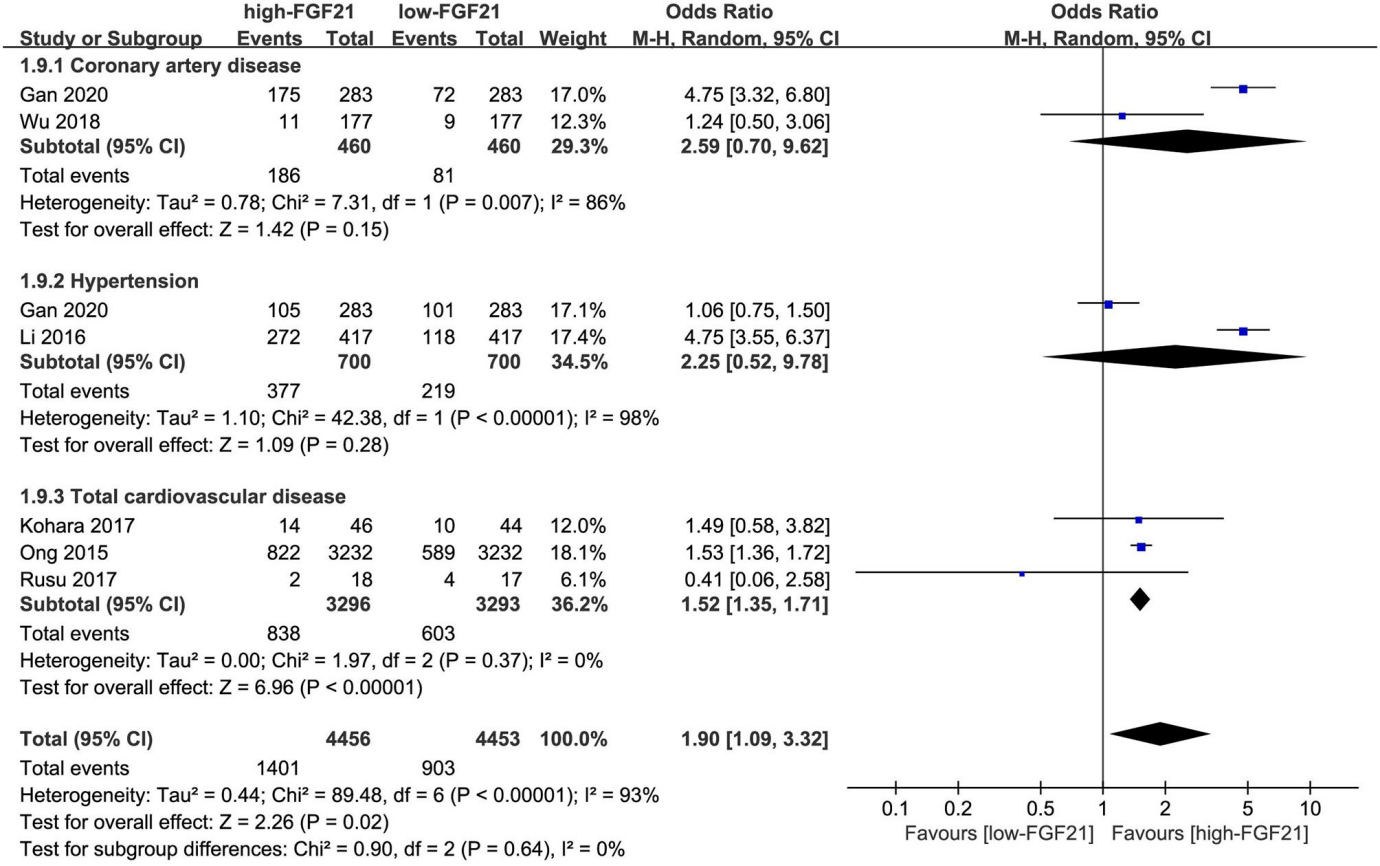
Forest plot of incidence of cardiovascular disease in individuals with different FGF21 levels. FGF21, fibroblast growth factor 21. Events refer to the numbers of corresponding disease that were diagnosed in each group.

The incidences of overall CVDs (OR = 2.10, 95% CI: 1.09–4.06, *p* = 0.03) and hypertension (OR = 4.75, 95% CI: 3.55–6.37, *p* < 0.001) were significantly higher in individuals with higher FGF21 levels (Figure 5). However, the difference in CAD incidence between individuals with different FGF21 levels was insignificant (*p* = 0.16).

## Discussion

FGF21 is involved in glucose and lipid metabolism. Studies have reported that FGF21 is increased in patients with non-alcoholic fatty liver disease, obesity, and diabetes (40). Therefore, FGF21 has been considered as a new biomarker for metabolic syndrome. For CVDs, metabolic syndrome is a well-known precursor (2), which suggests a possible link of FGF21 to CVDs. Our meta-analysis also demonstrated that FGF21 was increased in patients with CVDs, which may be caused by a compensatory response to underlying metabolic stress (41). Some studies reported the cutoff value (ranged from 123.0 to 321.5 pg/ml) of serum FGF21 as a predictor for CVDs according to Youden index (11, 14, 17, 25, 28, 29) (Supplementary Table 5).

Experimental data from animal studies revealed the association between FGF21 and CVDs. A study demonstrated that the administration of exogenous FGF21 significantly improved lipid metabolic disorders and reduced atherosclerotic plaque areas in animal models (42). In detail, FGF21 can reduce cholesterol synthesis by suppressing hepatic sterol regulatory element-binding protein 2 (43), increase lipoprotein catabolism in adipose tissue, and reduce hepatic very low-density lipoprotein export (44). These critical events are major contributors to FGF21's ability to enhance lipid profiles, which are partly mediated by CD36 and lipoprotein lipase. The mechanisms of atherosclerosis prevention induced by FGF21 may be associated with suppression of endoplasmic reticulum stress-mediated apoptosis (45). FGF21 can stimulate adiponectin secretion, which has a large effect on the inhibition of neointima formation through inhibition of canonical events, including macrophage infiltration, and smooth muscle proliferation (43, 45, 46). These events are generally believed to cause endoplasmic reticulum stress within the vasculature. The antioxidative function of FGF21 is also involved in the therapeutic effect in atherosclerotic Wistar rats, including increased levels of superoxide dismutase, reduced form of glutathione, and reduced form of malondialdehyde (47). Our previous study also demonstrated that FGF21 can protect vascular endothelial cells from H_2_O_2_-induced premature cell senescence and intracellular accumulation of reactive oxygen species through SIRT1 (48). In addition, Wang et al. also demonstrated the cardioprotective effects of FGF21 against doxorubicin-induced toxicity through the SIRT1/LKB1/AMPK pathway (49). These results indicate that FGF21 is not only a biomarker of CVDs but may also have a protective effect on the cardiovascular system. Lin et al. reported that serum FGF21 increased in apoE^−/−^ mice, and FGF21 deficiency enhanced atherosclerotic deterioration and mortality (43). Considering the mechanisms of atherosclerosis prevention induced by FGF21, the increased FGF21 may not be the basis for atherosclerotic pathogenesis while it may compensate for the increase during atherosclerosis and bring beneficial effects instead (5).

Based on strong preclinical evidence of the therapeutic effect of FGF21 in metabolic syndrome, clinical studies were conducted to evaluate the effect of FGF21 variants or analogs. PF-05231023, an FGF21 analog, was reported to have beneficial effects on body weight, lipoprotein profile, and adiponectin concentrations in overweight/obese subjects with type 2 diabetes (50). During the treatment of diabetic patients using at least 25 mg of PF-05231023 once a week, the minimum observed concentration was 0.768 μg/ml. It can also significantly lower triglycerides in the absence of weight loss in monkeys (51). LY2405319, another FGF21 analog, was found to improve lipid levels, lipoprotein profile, body weight, fasting insulin, and adiponectin levels in obese patients with type 2 diabetes in a randomized, placebo-controlled, double-blind clinical trial (52). During the treatment, average steady-state circulating plasma concentrations of LY2405319 on day 28 were 17.5 ± 4 ng/ml in subjects who received the 3-mg daily dose, and levels were 67.3 ± 25 ng/ml and 150 ± 49 ng/ml in subjects treated with 10 and 20 mg, respectively. Therefore, administration of exogenous supra-physiological doses of FGF21 variants or analogs may also provide a therapeutic benefit on CVDs. Unfortunately, no clinical study has directly evaluated the therapeutic effects of FGF21 analogs on CVDs at present. In our study, the risk of CVDs increased with FGF21, which also indicates the need for supra-physiological doses of FGF21 to achieve therapeutic efficacy. Clinical evidence is needed to investigate the role and the effective dose of FGF21 analogs in the prevention or treatment of CVDs as a novel therapeutic agent.

Arterial stiffness is one of the earliest indicators of changes in vascular wall structure and function, which can be assessed by using various indicators, including PWV, ABI, cardio-ankle vascular index, and cIMT (53). Increased arterial stiffness has a major effect on pulse pressure, which directly leads to abnormal blood pressure (54). A large number of clinical studies have demonstrated an association between arterial stiffness and atherosclerotic burden as well as between arterial stiffness and incident cardiovascular events. The association between arterial stiffness and atherosclerosis might be incidental, as the two processes occur at similar sites of the arterial tree and both progress with age, or might be explained by the impact of common risk factors, such as metabolic syndrome (55). However, the differences between atherosclerosis and arteriosclerosis should still be noted. Arteriosclerosis mainly refers to stiffening of the normally flexible walls due to loss of elasticity of the arterial musculature. The loss or disorder of elastin is the main reason for the thickening of the arterial walls. In atherosclerosis, there is a gradual increase in the deposition of plaque (consisting of calcium, white blood cells and clumps of platelets, cholesterol, and lipids) within the lumen leading to narrowing or complete blockage of the artery. The development of atherosclerosis is closely related to hyperglycemia and hyperlipidemia (56). Therefore, FGF21 has a stronger association with atherosclerosis than arteriosclerosis. In our meta-analysis, the association between FGF21 and vascular parameters was weak. Therefore, FGF21 may act as a CVD biomarker independent of vascular parameters, such as PWV, ABI, cIMT, and BP.

Several limitations of our meta-analysis must be taken into consideration. First, due to different patient selection criteria and baseline characteristics, such as age and gender, significant heterogeneity was observed in our meta-analysis. Second, the definition of CVDs and CHD varied among the included studies. Third, the follow-up time varied among each included study, which may bring bias in the estimation of pooled HR of FGF21 concentration and CVDs. Fourth, our meta-analysis only included CHD, hypertension, and overall CVDs. Studies on other CVDs such as peripheral arterial disease, rheumatic heart disease, atrial fibrillation, cerebral infarction, cardiomyopathy, or microvascular disease were not included. Fifth, original data reported in articles were transformed for meta-analysis, especially for data of median value with quartile. Those original data with skewed distribution may not be suitable for meta-analysis. Sixth, the threshold for FGF21 level classification varied among studies. A standard FGF21 classification threshold needs to be established before clinical use for CVD risk assessment. Several modalities were applied to reduce these limitations. First, we conducted a systematic, comprehensive search in two databases. Second, we strictly stipulated the inclusion criteria, eliminating the bias caused by some potential confounding factors, and the data were independently extracted by two reviewers. Third, we conducted a subgroup analysis of specific CVDs.

## Conclusion

High-level serum FGF21 concentration is closely associated with an increased risk of CVDs, which may be independent of vascular parameters, including cIMT, PWV, ABI, and BP. A standard FGF21 classification threshold needs to be established before clinical use for CVD risk assessment.

## Data Availability Statement

The original contributions presented in the study are included in the article/Supplementary Material, further inquiries can be directed to the corresponding author/s.

## Author Contributions

CZ and LZ: conceptualization. YZ: methodology. JY and NY: investigation. ZQ and HN: data curation. ZY, DY, and XW: formal analysis. YZ, LR, and YH: writing, review, and editing of the manuscript. All authors contributed to the article and approved the submitted version.

## Conflict of Interest

The authors declare that the research was conducted in the absence of any commercial or financial relationships that could be construed as a potential conflict of interest.

## Publisher's Note

All claims expressed in this article are solely those of the authors and do not necessarily represent those of their affiliated organizations, or those of the publisher, the editors and the reviewers. Any product that may be evaluated in this article, or claim that may be made by its manufacturer, is not guaranteed or endorsed by the publisher.
