# Supplementary material for: High-Level Serum Fibroblast Growth Factor 21 Concentration Is Closely Associated With an Increased Risk of Cardiovascular Diseases: A Systematic Review and Meta-Analysis
Source: Front Cardiovasc Med. 2021 Aug 26;8:705273. doi: 10.3389/fcvm.2021.705273 (PMC8427036; doi:10.3389/fcvm.2021.705273)
Supplement: Supplementary file 1 [file Data_Sheet_1.DOCX]

Supplementary Material

# Supplementary Tables and Figures

## Supplementary Tables

| Supplementary Table 1 Patient selection criteria of all included studies   \| Study \| Patient selection criteria \| \| --- \| --- \| \| Wu 2021 \| **Inclusion**: (1) clinically diagnosed with myocardial infarction (>3 months); (2) demonstrated at least one coronary artery stenosed by >50% by coronary angiography; (3) demonstrated coronary artery stenosis or myocardial infarction after chest pain; or (4) had undergone coronary artery bypass graft or percutaneous coronary intervention (>3 months). **Exclusion**: (1) a history of depression or other psychiatric disorders, and on anti-depressant or psychotropic medication; (2) acute myocardial infarction during hospitalization (manifested by electrocardiographic changes and/or elevated myocardial enzymes); (3) myocardial infarction or cardiac surgery in the past 3 months; (4) an acute infectious disease in the month prior to enrollment; (5) other severe cardiovascular diseases (e.g., acute pericarditis, myocarditis, end-stage heart failure, and secondary heart disease); (6) diseases seriously affecting life expectancy (e.g., connective tissue disease, cancer, drug abuse, and dementia); (7) pregnancy; (8) recent major stressful life events; or (9) an inability to complete the depression scale assessment or blood sampling. \| \| Wu 2020 \| **Cross-sectional cohort: Inclusion:** patients with T2DM without NAFLD. **Exclusion:** (1) hepatic fat fraction ≥5.56%; (2) clinical symptoms of atherosclerosis or the presence of CVD, including angina, myocardial infarction, heart failure, stroke, transient ischemic attack, or a prior invasive cardiovascular procedure; (3) age younger than 45 or older than 75 years; (4) liver or kidney dysfunction (alanine transaminase >40 U/L or eGFR <30 mL/min per 1.73 m^2^); (5) alcoholism (≥140 g per week for men or ≥70 g per week for women); (6) known history of viral hepatitis, autoimmune hepatitis, severe infection, or cancer; and (7) current use of lipid-lowering drugs such as fenofibrate (which significantly increases hepatic FGF21 expression).  **Prospective cohort: Exclusion:** Subjects with cancer, severe disability or psychiatric disturbance. \| \| Gan 2020 \| **Inclusion:** aged more than 18 years, CAC detected by CCTA, life expectancy more than at least one year, ability to complete yearly follow-up for at least one year, no previous history of PCI, and signed the informed content profile. **Exclusion:** malignant tumor, connective tissue diseases, active liver diseases; eGFR less than 60 mL/min/1.73 m2 or dialysis; previous history of PCI, cardiac surgery, or other micro traumatic cardiac intervention; and emergent PCI administered at the same time of enrollment. \| \| Lee 2020 \| Exercise for Life across Asia (ELIXA) cohort. Patients’ selection criteria were not provided. \| \| Yamam-  oto2020 \| **Inclusion:** aged more than 60 years. **Exclusion:** Patients with apparent infection, active neoplastic diseases, and acute kidney injury. \| \| Sunaga 2019 \| **Inclusion:** patients with a final diagnosis of AMI patients with a final diagnosis of AMI**. Exclusion:** patients with end-stage renal disease receiving hemodialysis and those who were admitted more than 24 hours after the onset of chest pain \| \| Ong 2019 \| Multi-Ethnic Study of Atherosclerosis: 6,500 men and women, in equal numbers, who are aged 45–84 years and free of clinical CVD at baseline, including four racial/ethnic groups from six US communities. https://www.mesa-nhlbi.org/aboutMESAOverviewProtocol.aspx \| \| Basurto 2019 \| **Inclusion:** i) women, ii) aged 45–60 years, and iii) without clinical evidence of CVD. **Exclusion:** i) the presence of T2DM, renal or liver failure, ii) current chronic infections, endocrine, or blood disorders, and/or iii) a history of CVD or thrombosis. \| \| Yafei 2019 \| **Inclusion:** patients with T2DM. **Exclusion:** patients with overt cardiovascular disease or history of revascularization or clinical evidence of peripheral arterial diseases; patients with endocrinal or metabolic diseases, type 1 diabetes, gestational diabetes, renal or hepatic impairment. \| \| Ong 2019A \| **Inclusion:** men and women 35 to 75 years of age who had previous myocardial infarction, previous or current angina with objective evidence of atherosclerotic CHD, or a history of coronary revascularization. **Exclusion:** hypersensitivity to statins; active liver disease or hepatic dysfunction defined as alanine aminotransferase or aspartate aminotransferase >1.5 times the upper limit of normal; women who are pregnant or breastfeeding; patients with nephrotic syndrome; uncontrolled diabetes mellitus; uncontrolled hypothyroidism; uncontrolled hypertension (as defined by the investigator) at the screening visit; a MI, coronary revascularization procedure or severe/unstable angina within 1 month of screening; any planned surgical procedure for the treatment of atherosclerosis; an ejection fraction <30%; hemodynamically important valvular disease; gastrointestinal disease limiting drug absorption or partial ileal bypass; any nonskin malignancy, malignant melanoma or other survival-limiting disease; unexplained creatine phosphokinase levels >6 times the upper limit of normal; concurrent therapy with long-term immunosuppressants; concurrent therapy with lipid-regulating drugs not specified as study treatment in the protocol; history of alcohol abuse; and participation in another clinical trial concurrently or within 30 days before screening. \| \| Chen 2018 \| **Inclusion:** aged over 18 years, AMI patients and patients with chest pain, whose coronary angiography and Troponin I (cTn I) were negative. **Exclusion:** the presence of acute or chronic viral hepatitis, fatty liver, drug or alcoholic-induced liver disease, a history of valvular heart disease, cardiomyopathy, myocarditis, congenital heart disease, peripheral vascular disease, or infective endocarditis, or of a combination of these ailments, acute or chronic kidney diseases (CKD stage 3-5), total parenteral nutrition, alcoholism, hyper-or hypothyroidism, cancer, current treatment with systemic corticosteroid, fenofibrate, metformin or thiazolidane treatment. \| \| Cheng 2018 \| **Inclusion:** (i) 66 subjects with SAP, diagnosed as paroxysmal exertional chest discomfort that was accompanied with the changes of electrocardiogram in an exercise test; (ii) 76 subjects with UAP, presented as chest pain at rest or aggravated effort type angina within 1 month with the changes of definite ischemic electrocardiogram or recurrent angina pectoris; and (iii) 55 control subjects with normal coronary artery findings and no changes of ECG ischemic ST-T. **Exclusion:** (i) patients with congenital heart disease, myocarditis, thromboembolism, cardiomyopathy, collagen disease; (ii) severe kidney and liver diseases; (iii) any malignant diseases; and (iv) some inflammatory diseases, such as septicemia and septicopyemia. \| \| Wu 2018 \| **Inclusion:** subjects with stable CAD admitted for elective CAG and/or PCI. **Exclusion:** patients with stage 5 CKD, defined as creatinine clearance <15 mL/min/1.73 m2, and those with pre-existing dialysis requirements were excluded from the analysis; patients under the treatment of fenofibrate, which was known to be a PPARα agonist and may potentially affect circulating FGF21 concentrations \| \| Shen 2018 \| **Inclusion:** subjects for examination by coronary arteriography. **Exclusion:** the presence of acute or chronic viral hepatitis, drug-induced or alcoholic liver diseases, alcoholism (a total of ≥ 140 g per week by a male adult or a total of ≥ 70 g per week by a female), total parenteral nutrition, chronic kidney diseases, hyper- or hypothyroidism, cancer, and current treatment with systemic corticosteroids, incomplete anthropometric or laboratory data \| \| Trakarnvanich 2017 \| **Inclusion:** patients ages >18 years who had received a renal transplant >1 month before enrollment. **Exclusion:** subjects with an eGFR <15 mL/min/1.73m^2^, limb amputation, and/or cardiac arrhythmia \| \| Shen 2017 \| **Inclusion:** subjects for examination by coronary arteriography. **Exclusion:** the presence of acute or chronic viral hepatitis, drug-induced or alcoholic liver diseases, alcoholism (a total of ≥ 140 g per week by a male adult or a total of ≥ 70 g per week by a female), total parenteral nutrition, chronic kidney diseases, hyper- or hypothyroidism, cancer, and current treatment with systemic corticosteroids, incomplete anthropometric or laboratory data \| \| Lee 2017 \| **Inclusion:** subjects with T2DM. **Exclusion:** subjects who were on fibrates or had known CVD at baseline. \| \| Kohara 2017 \| **Inclusion:** chronic hemodialysis patients. **Exclusion:** active malignancy, pulmonary disease, severe infectious disease, peritoneal dialysis, death within three months after study entry, and failure to cooperate with the study or provide consent to participate. \| \| Rusu 2017 \| **Inclusion:** prevalent hemodialysis patients, age >18 years, duration of maintenance hemodialysis at least 6 months (hemodialysis vintage). **Exclusion:** acute inflammation, neoplasia, hepatitis virus infection and any other serious chronic or acute diseases requiring treatment. All patients were on thrice weekly hemodialysis (4–5 h) regimen. Patients’ demographic data, etiology of end-stage renal disease (ESRD), hemodialysis vintage, comorbidity conditions (diabetes, hypertension, smoking status), antihypertensive treatment, statins, antiplatelet therapy, erythropoietin treatment and intravenous iron upon enrollment were obtained from medical documents. \| \| Li 2016 \| **Inclusion:** (1) a history of or newly diagnosed CAD, (2) age ≥ 40 years at baseline survey, and (3) having stayed in Guangdong province at least 5 years. **Exclusion:** (1) age < 40 or >85 years, (2) other cardiac origins (aortic valve stenosis or insufficiency, aortic dissection, acute pericarditis, rheumatic coronaritis, hypertrophic cardiomyopathy, syphilitic aortic regurgitation and cardioneurosis) and noncardiac (respiratory, gastrointestinal, or musculoskeletal) chest pain, (3) severe liver and/or kidney failure, and (4) a history of or newly diagnosed autoimmune disease or thyroid disorder. \| \| Zhang 2015 \| **Inclusion:** AMI patients aged over 18 years old. **Exclusion:** (1) allergy to or inability to tolerate statins; (2) stroke or a history of visceral bleeding disorders in the previous 6 months; (3) severe kidney disease and/or coagulation abnormalities; (4) a history of valvular heart disease, cardiomyopathy, myocarditis, congenital heart disease, peripheral vascular disease, or infective endocarditis, or of a combination of these ailments; (5) Stage 3–5 CKD; (6) cancer or life expectancy of no more than 1 year; and (7) chronic heart failure and other diseases that adversely affect short-term prognosis. \| \| Xiao 2015 \| **Inclusion:** patients who were diagnosed within 1 year with type 2 diabetes. **Exclusion:** clinical symptoms of atherosclerosis; presence of CVD (a history of physician-diagnosed myocardial infarction, angina, heart failure, stroke, or transient ischemic attack or who had undergone an invasive cardiovascular procedure); diabetic nephropathy or diabetic retinopathy; or severe liver and kidney dysfunction. \| \| Kim 2015 \| **Inclusion:** 18 to 80 years old patients who underwent coronary angiography due to chest pain. **Exclusion:** 1) severe liver and renal dysfunction (AST and ALT of three times above the normal limit, creatinine > 2 mg/dL), 2) heart failure (left ventricular ejection fraction < 40%), 3) a history of acute myocardial infarction, 4) a history of coronary artery disease underwent coronary angiography, 5) a recent history of hematologic disease, 6) connective tissue disease, 7) valvular heart disease, and 8) pregnancy. \| \| Ong 2015 \| **Inclusion:** • male or female, aged 50–75 years inclusive • non-insulin dependent diabetes mellitus (type 2) with age at diagnosis >35 years (currently using any of diet, tablets or insulin); for Maori, Pacific Islanders, Australian Aborigines and Torres Strait Islanders, the eligible age of diagnosis was >25 years, provided there had been at least 1 year of treatment without insulin • on the basis of diabetes, considered to be at higher risk for coronary heart disease than the general population • no clear indication for any cholesterol-lowering treatment: the patient was not already taking any cholesterol-lowering drug and neither the patient nor the patient's doctor considered there to be any definite need to do so • total cholesterol level 3 to 6.5 mmol/L, plus either  a total cholesterol-to-HDL cholesterol ratio of ≥ 4.0  a blood triglyceride level >1.0 mmol/L • no clear contraindication to study therapy in the view of the treating physician • no other predominant medical problem that might limit compliance with 5 years of study treatment or compromise long-term participation and clinic attendance in the trial. **Exclusion:** • serum triglyceride >5 mmol/L in the baseline visit fasting blood sample • concurrent treatment with any other lipid-lowering agent • serum creatinine >130 μmol/L • known chronic liver disease, transaminases >2 × upper limit of normal or symptomatic gall-bladder disease • myocardial infarction or hospital admission for unstable angina within 3 months • female, of child-bearing potential, unless sterilized or on reliable approved methods of contraception, including oral contraceptives • concurrent cyclosporin treatment (or a condition likely to result in organ transplantation and the need for cyclosporin during the next 5 years) • known allergy to any fibrate drug or known photosensitivity • unwilling or unable to consent to enter the study, with the understanding that follow-up was planned to continue for more than 5 years. \| \| Shen 2013 \| **Inclusion:** subjects for examination by coronary arteriography. **Exclusion:** the presence of acute or chronic viral hepatitis, drug-induced or alcoholic liver diseases, alcoholism (a total of ≥ 140 g per week by a male adult or a total of ≥ 70 g per week by a female), total parenteral nutrition, chronic kidney diseases, hyper- or hypothyroidism, cancer, and current treatment with systemic corticosteroids, incomplete anthropometric or laboratory data. \| \| Chow 2013 \| Subjects who underwent carotid IMT measurement. \| \| Lipińska 2013 \| **Inclusion:** T2DM patients on stable medications for at least 3 months. **Exclusion:** type 1 diabetes, type 2 diabetes with acute infection or recent (<6 months) myocardial infarction, unstable angina, stroke, sustained hypertension, cancer, severe kidney and liver diseases, asthma, tumours, connective tissue diseases and other known acute illnesses. \| \| Lee 2014 \| Patients who had undergone a cardiac evaluation with 64-slice multidetector computed tomography (MDCT) either as a routine physical examination or for cardiac evaluation in high-risk patients with diabetes and hypertension, dyslipidaemia, history of smoking, strong family history of CAD, previously known or suspected atherosclerotic disease other than CAD and/or microalbuminura. \| \| Semba 2014 \| Male between the ages of 17 and 96. (Normal Human Aging: The Baltimore Longitudinal Study of Aging) \| \| Lin 2010 \| **Inclusion:** individuals suffered from angina pectoris or myocardial infarctions. **Exclusion:** The CHD patients who received medication within at least one year for lipid lowering, anti-hypertension and diabetic treatment. \|   T2DM: type 2 diabetes mellitus; NAFLD: nonalcoholic fatty liver disease; CVD: cardiovascular disease; FGF21: fibroblast growth factor 21; CAC: artery calcification; PCI: percutaneous coronary intervention; AMI: acute myocardial infarction; SAP: stable angina pectoris; CAD: coronary artery disease; eGFR: estimated glomerular filtration rate; CKD: chronic kidney disease; CAG: coronary angiography; IMT: intima-media thickness; CHD: coronary heart disease;  Supplementary Table 2. Quality assessment of Cohort studies by Newcastle–Ottawa Scale. | | | | | | | | | | | | |
| --- | --- | --- | --- | --- | --- | --- | --- | --- | --- | --- | --- | --- | --- | --- | --- | --- | --- | --- | --- | --- | --- | --- | --- | --- | --- | --- | --- | --- | --- | --- | --- | --- | --- | --- | --- | --- | --- | --- | --- | --- | --- | --- | --- | --- | --- | --- | --- | --- | --- | --- | --- | --- | --- | --- | --- | --- | --- | --- | --- | --- | --- | --- | --- | --- | --- | --- | --- | --- | --- | --- | --- | --- | --- | --- |
| Included Studies | Selection | | | |  | Comparability | |  | Outcome | | | Total Scores |
|  | A | B | C | D |  | A1 | B1 |  | A2 | B2 | C2 |  |
| Wu 2020 | 1 | 1 | 1 | 1 |  | 1 | 1 |  | 1 | 1 | 0 | 8 |
| Gan 2020 | 1 | 1 | 1 | 1 |  | 1 | 1 |  | 1 | 1 | 1 | 9 |
| Ong 2019 | 1 | 1 | 1 | 1 |  | 1 | 1 |  | 1 | 1 | 1 | 9 |
| Ong 2019A | 1 | 1 | 1 | 1 |  | 1 | 1 |  | 1 | 1 | 1 | 9 |
| Chen 2018 | 1 | 1 | 1 | 1 |  | 1 | 1 |  | 1 | 0 | 1 | 8 |
| Wu2018 | 1 | 1 | 1 | 1 |  | 1 | 1 |  | 1 | 1 | 1 | 9 |
| Shen 2018 | 1 | 1 | 1 | 1 |  | 1 | 1 |  | 1 | 1 | 1 | 9 |
| Shen 2017 | 1 | 1 | 1 | 1 |  | 1 | 1 |  | 1 | 1 | 1 | 9 |
| Lee 2017 | 1 | 1 | 1 | 1 |  | 1 | 1 |  | 1 | 1 | 1 | 9 |
| Kohara 2017 | 1 | 1 | 1 | 1 |  | 1 | 1 |  | 1 | 1 | 1 | 9 |
| Li 2016 | 1 | 1 | 1 | 1 |  | 1 | 1 |  | 1 | 1 | 1 | 9 |
| Ong 2015 | 1 | 1 | 1 | 1 |  | 1 | 1 |  | 1 | 1 | 1 | 9 |
| Lee 2014 | 1 | 1 | 1 | 1 |  | 1 | 1 |  | 1 | 1 | 1 | 9 |
| Lipińska 2013 | 1 | 1 | 1 | 1 |  | 1 | 1 |  | 1 | 0 | 1 | 8 |

A: Representativeness of exposed cohort. B: Representativeness of unexposed cohort. C: Ascertainment of exposure (If the exposure data was obtained from prescription database or medical record). D: Outcome was not present at start. A1: Important factor (If adjusted for the age, a point was assigned.) B1: Additional factor (If adjusted for any other additional factors.) A2: Assessment of outcome. B2: Exposure Follow-up for outcomes. C2: Rate of follow-up

| Supplementary Table 3. Quality assessment of Case-control studies by Newcastle–Ottawa Scale. | | | | | | | | | | | | |
| --- | --- | --- | --- | --- | --- | --- | --- | --- | --- | --- | --- | --- |
| Included Studies | Selection | | | |  | Comparability | |  | Outcome | | | Total Scores |
|  | A | B | C | D |  | A1 | B1 |  | A2 | B2 | C2 |  |
| Semba 2014 | 1 | 1 | 1 | 1 |  | 1 | 1 |  | 1 | 1 | 1 | 9 |

A: Adequacy of case definition. B: Representativeness of the cases. C: Selection of controls. D: Definition of controls. A1: Important factor (If adjusted for the age, a point was assigned.) B1: Additional factor (If adjusted for any other additional factors.) A2: Ascertainment of exposure. B2: Same method of ascertainment for cases and controls. C2: Non-response rate.

Supplementary Table 4. Quality assessment of Cross-section studies by using the Joanna Briggs Institute Checklist

| Included Studies | JBI quality assessment criteria’s | | | | | | | | Total score |
| --- | --- | --- | --- | --- | --- | --- | --- | --- | --- |
|  | ① | ② | ③ | ④ | ⑤ | ⑥ | ⑦ | ⑧ |  |
| Wu 2021 | Y | Y | Y | Y | Y | Y | Y | Y | 8 |
| Wu 2020 | Y | Y | Y | Y | Y | Y | Y | Y | 8 |
| Lee 2020 | Y | Y | Y | Y | Y | Y | Y | Y | 8 |
| Yamamoto 2020 | Y | Y | Y | Y | Y | Y | Y | Y | 8 |
| Sunage 2019 | Y | Y | Y | Y | Y | Y | Y | Y | 8 |
| Basurto 2019 | Y | Y | Y | Y | Y | Y | Y | Y | 8 |
| Yafei 2019 | Y | Y | Y | Y | Y | Y | Y | Y | 8 |
| Cheng 2018 | Y | Y | Y | Y | Y | Y | Y | Y | 8 |
| Trakarnvanich 2017 | Y | Y | Y | Y | Y | Y | Y | Y | 8 |
| Rusu 2017 | Y | Y | Y | Y | Y | Y | Y | Y | 8 |
| Zhang 2015 | Y | Y | Y | Y | Y | Y | Y | Y | 8 |
| Xiao 2015 | Y | Y | Y | Y | Y | Y | Y | Y | 8 |
| Kim 2015 | Y | Y | Y | Y | Y | Y | Y | Y | 8 |
| Shen 2013 | Y | Y | Y | Y | Y | Y | Y | Y | 8 |
| Chow 2013 | Y | Y | Y | Y | Y | Y | Y | Y | 8 |
| Lin 2010 | Y | Y | Y | Y | Y | Y | Y | Y | 8 |

Y: Yes, N: No, U: Unclear, NA: Not applicable. ①: Were the criteria for inclusion in the sample clearly defined? ②: Were the study subjects and the setting described in detail? ③: Was the exposure measured in a valid and reliable way? ④: Were objective, standard criteria used for measurement of the condition? ⑤: Were confounding factors identified? ⑥: Were strategies to deal with confounding factors stated? ⑦: Were the outcomes measured in a valid and reliable way? ⑧: Was appropriate statistical analysis used?

Supplementary Table 5 Cutoff value of serum fibroblast growth factor 21 as a predictor for cardiovascular diseases

| Study | Disease | Cutoff value^#^ (pg/ml) | Sensitivity^#^ (%) | Specificity^#^ (%) |
| --- | --- | --- | --- | --- |
| Wu2020 | ischemic heart disease | 232.0 | - | - |
| Yafei2019 | subclinical atherosclerosis | 184 | 66.7 | 66.7 |
| Chen2018 | major adverse cardiovascular events | 123.0 | 80.0 | 50.4 |
| Shen2018 | coronary artery disease | 239.7 | 71.2 | 59.5 |
| Shen2017 | left ventricular dysfunction | 321.5 | 70.6 | 59.2 |
| Lee2017 | incident coronary heart disease | 206.2 | - | - |

^#^ Obtained from the Youden index.


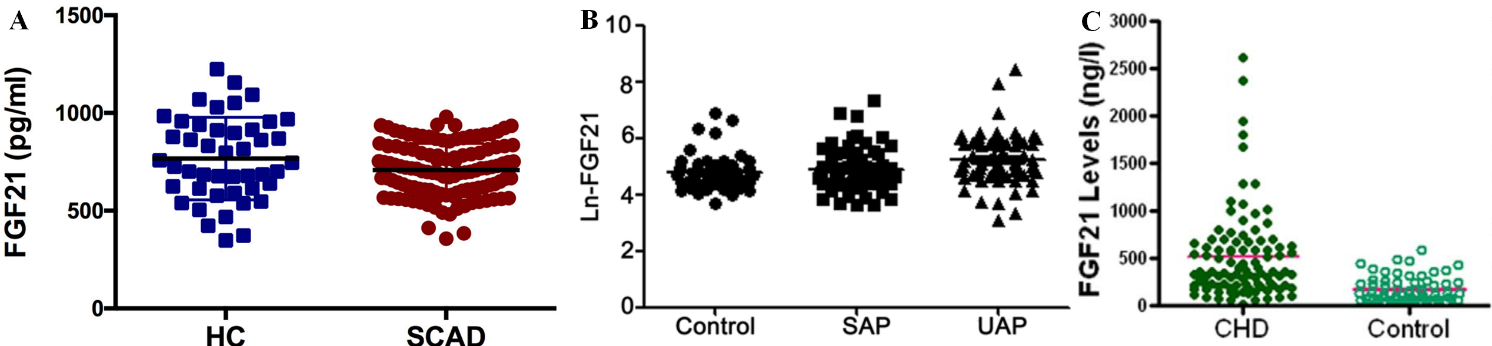


**Supplementary Figure 1**. Serum fibroblast growth factor 21 concentrations in patients with or without CVD reported in three independent studies~~.~~: (A) Wu, Y., Z. Chen, J. Duan, K. Huang, B. Zhu and L. Yang, et al., Serum Levels of FGF21, beta-Klotho, and BDNF in Stable Coronary Artery Disease Patients With Depressive Symptoms: A Cross-Sectional Single-Center Study. Front Psychiatry, 2020. 11: p. 587492. (B) Chen, H., N. Lu and M. Zheng, A high circulating FGF21 level as a prognostic marker in patients with acute myocardial infarction. Am J Transl Res, 2018. 10(9): p. 2958-2966. (C) Lin, Z., Z. Wu, X. Yin, Y. Liu, X. Yan and S. Lin, et al., Serum levels of FGF-21 are increased in coronary heart disease patients and are independently associated with adverse lipid profile. PLoS One, 2010. 5(12): p. e15534. FGF21: fibroblast growth factor 21; HC: health control; SCAD: stable coronary artery disease; SAP: stable angina pectoris; UAP: un stable angina pectoris; CHD: coronary heart disease.


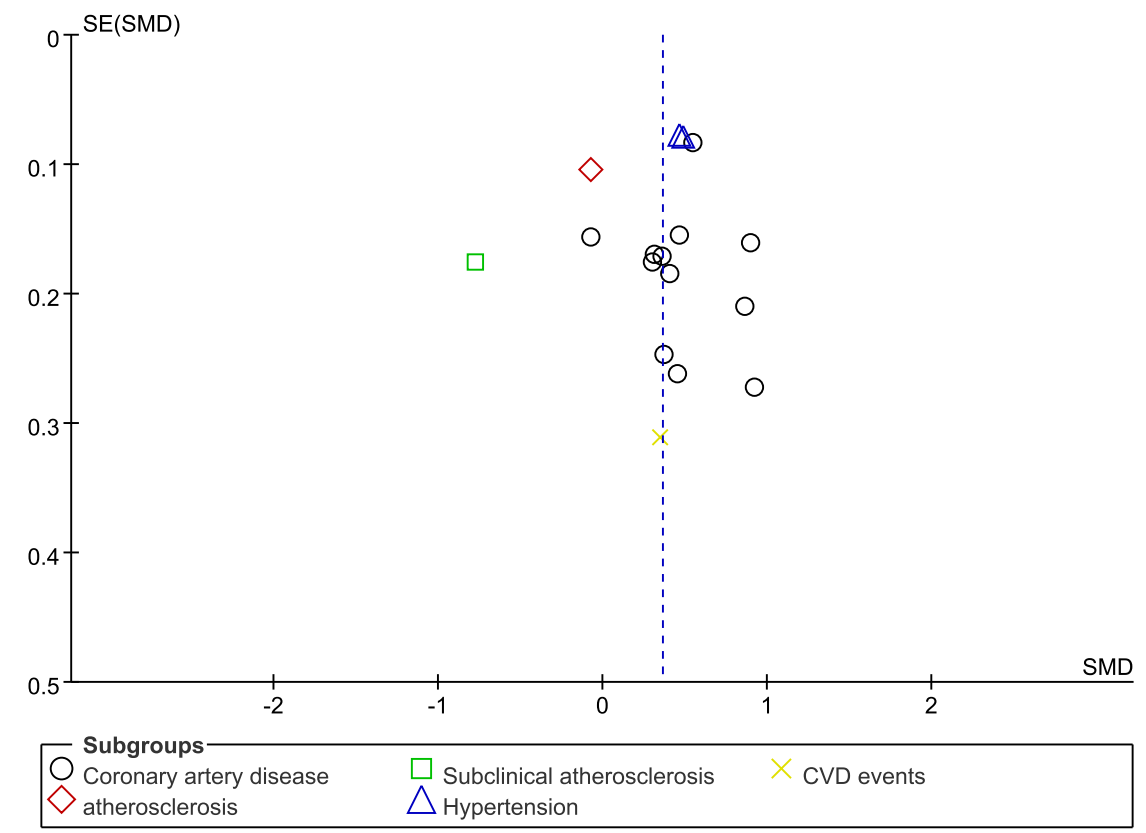


**Supplementary Figure 2**. The funnel plot of the differences of serum fibroblast growth factor 21 concentration in individuals with or without CVDs. SMD: standard mean difference; CVD: cardiovascular disease.


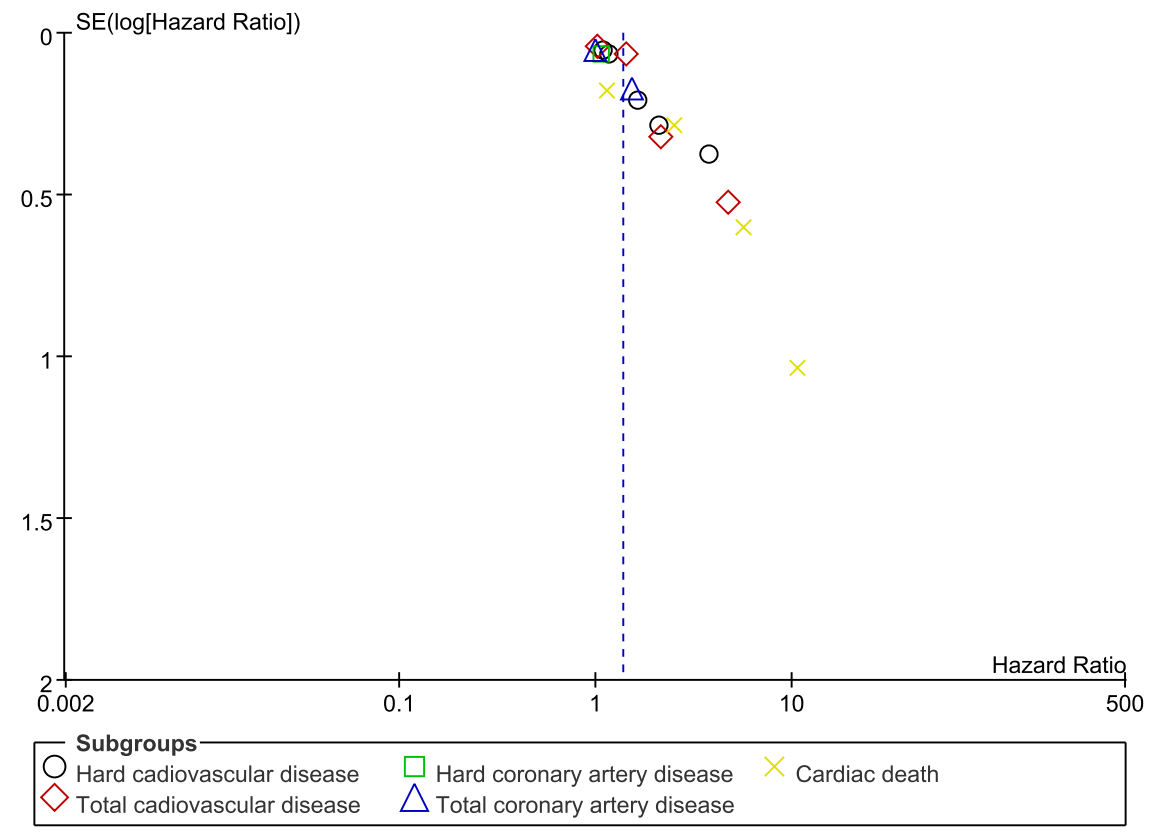


**Supplementary Figure 3.** The funnel plot of the association between serum fibroblast growth factor 21 concentration and prevalence of cardiovascular disease.
